# Supplementary material for: Asynchronous magnetic resonance elastography: Shear wave speed reconstruction using noise correlation of incoherent waves
Source: Magn Reson Med. 2022 Oct 27;89(3):990–1001. doi: 10.1002/mrm.29502 (PMC9792433; doi:10.1002/mrm.29502)
Supplement: Supplementary file 1 — DATA S1 MATLAB code used to generate the numerical simulations of Figure 1 [file MRM-89-990-s002.zip › k-Wave/helpfiles/kspaceLineRecon.html]

kspaceLineRecon :: Functions (k-Wave)


# kspaceLineRecon

2D linear FFT reconstruction.

## Syntax

```
p_xy = kspaceLineRecon(p_ty, dy, dt, c)
p_xy = kspaceLineRecon(p_ty, dy, dt, c, ...)
```

## Description

`kspaceLineRecon` takes an acoustic pressure time-series `p_ty` recorded over an evenly spaced array of sensor points on a line, and constructs an estimate of the initial acoustic pressure distribution that gave rise to those measurements using an algorithm based on the FFT. The input `p_ty` must be indexed `p_ty(time step, sensor y position)`, where the sensor spacing is given by `dy`, the temporal spacing given by `dt`, and the sound speed in the propagation medium (which is assumed to be acoustically homogeneous) is given by `c`. The output `p_xy` is indexed as `p_xy(x position, y position)`.

The code uses a k-space algorithm which performs (1) a Fourier transform on the data `p_ty` along both `t` and `y` dimensions (into wavenumber-frequency space, where the wavenumber component is along the detector line), (2) a mapping, based on the dispersion relation for a plane wave in an acoustically homogeneous medium, from wavenumber-frequency space to wavenumber-wavenumber space (where the second component is orthogonal to the detector line), and finally (3) an inverse Fourier transform back from the wavenumber domain to the spatial domain. The result is an estimate of the initial acoustic pressure distribution from which the acoustic waves originated.

Steps (1) and (3) can be performed efficiently using the fast Fourier transform (FFT); they are therefore fastest when the number of samples and number of detector points are both powers of 2. The mapping in step (2) requires an interpolation of the data from an evenly spaced grid of points in the wavenumber-frequency domain to an evenly-spaced grid of points in the wavenumber-wavenumber domain. The option `'Interp'` may be used to choose the interpolation method.

The physics of photoacoustics requires that the acoustic pressure is initially non-negative everywhere. The estimate of the initial pressure distribution generated by this code may have negative regions due to artefacts arising from differences between the assumed model and the real situation, e.g., homogeneous medium vs. real, somewhat heterogeneous, medium; infinite measurement surface vs. finite-sized region-of-detection, etc. A positivity (or non-negativity) condition can be enforced by setting the optional `'PosCond'` to `true` which simply sets any negative parts of the final image to zero.

## Inputs

|  |  |
| --- | --- |
| `p_tx` | pressure time-series recorded over an evenly spaced array of sensor points on a line (indexed as t, y) |
| `dy` | spatial step [m] |
| `dt` | time step [s] |
| `c` | acoustically-homogeneous sound speed [m/s] |

## Optional Inputs

Optional 'string', value pairs that may be used to modify the default computational settings.

| Input | Valid Settings | Default | Description |
| --- | --- | --- | --- |
| `'DataOrder'` | `'ty'` `'yt'` | `'ty'` | String input which sets the order of the input data. |
| `'Interp'` | *(string of interpolation type)* | `'*nearest'` | String input controlling the interpolation method used by `interp2` in the reconstruction. |
| `'Plot'` | *(Boolean scalar)* | `false` | Boolean controlling whether a plot of the reconstructed estimate of the initial acoustic pressure distribution is produced. |
| `'PosCond'` | *(Boolean scalar)* | `false` | Boolean controlling whether a positivity condition is enforced on the reconstructed estimate of the initial acoustic pressure distribution. |

## Outputs

|  |  |
| --- | --- |
| `p_xy` | estimate of the initial acoustic pressure distribution (indexed as x, y) |

## Examples

- 2D FFT Reconstruction For A Line Sensor
- 2D Time Reversal For A Line Sensor

## See Also

`interp2`, `kspacePlaneRecon`, `kWaveGrid`
